# The Process of Developing a WHO Dementia-Friendly Community Global Toolkit: Input From Multiple Stakeholders

**DOI:** 10.1093/geroni/igaa057.2449

**Published:** 2020-12-16

**Authors:** Fei Sun, Katrin Seeher, Stéfanie Fréel, Tarun Dua

**Affiliations:** 1 Michigan State University, East Lansing, Michigan, United States; 2 WHO, Geneva, Geneve, Switzerland

## Abstract

This study examined the process that the Department of Mental Health and Substance Abuse of WHO used to develop a global toolkit for dementia friendly initiatives (DFI). Data were collected through a mix-method approach consisting of individual interviews of 20 DFI leaders, four focus group interviews of persons living with dementia (PWD), three group interviews of professionals, and an online survey of 129 participants from 46 countries. Data from multiple sources were examined. The meaning of DFIs centered on the needs of PWD, multi-sector collaboration, and physical and social environmental changes. Over 70% participants in the survey reported their DFIs targeted PWD and included PWD as important partners. The EASTT model can be used to summarize DFI strategies including Education, Advocacy, Support, Training and Transforming environment. Countries advanced in DFI tended to focus on enhancing professional capacity and environmental adaptation, while countries launching DFI appeared to prioritize dementia awareness campaigns.

